# Sex-Related Microglial Perturbation Is Related to Mitochondrial Changes in a Model of Alzheimer’s Disease

**DOI:** 10.3389/fncel.2022.939830

**Published:** 2022-07-05

**Authors:** Eoin O’Neill, Virginia Mela, Aline Sayd Gaban, Sibylle Bechet, Aoife McGrath, Aife Walsh, Allison McIntosh, Marina A. Lynch

**Affiliations:** Trinity College Institute of Neuroscience, Trinity College Dublin, Dublin, Ireland

**Keywords:** Alzheimer’s disease, microglia, mitochondrial dysfunction, iron accumulation, sex-related differences

## Abstract

Many studies implicate microglia in the pathogenesis of Alzheimer’s disease (AD) but precisely how these cells make their impact has not been determined to date. One contributory factor is likely to be the enhanced production of inflammatory mediators and it is now known that microglia with this secretory phenotype exhibit other adaptations including in their morphology, function, and metabolism. AD, like many neurological disorders, demonstrates a sex bias and recent evidence indicates that the sexual dimorphism in microglial function, which has been recognized for many years in early development, persists into adulthood and aging. Here, we demonstrate sex-related differences in microglia from post mortem tissue of male and female AD patients and a marked increase in the number of dystrophic and rod-shaped microglia in tissue from female AD patients compared with males. Furthermore, there was an increase in iron-laden microglia in tissue from female AD patients and this has been reported to reflect mitochondrial changes. To address this further, we assessed changes in microglia from male and female APP/PS1 mice and demonstrate that iron accumulation in microglia is increased to a greater extent in tissue prepared from females compared with males. This was associated with altered expression of genes coding for proteins that modulate mitochondrial function. The findings suggest that sex-related differences in the severity and perhaps incidence of AD may, at least in part, arise from sexual dimorphism in microglia.

## Introduction

Recent analysis using bulk and single cell RNA-sequencing have identified microglial signatures that describe the homeostatic state and, in aging and disease models, several non-homeostatic states. Some studies have reported that transcripts which define the homeostatic state of microglia are downregulated, while those that reflect immune activation and inflammation are upregulated, in aged compared with young mice (Hickman et al., 2013; Holtman et al., 2015; Krasemann et al., 2017; Kang et al., 2018; Hammond et al., 2019). Similar up- and down-regulated transcripts were observed in models of disease, particularly Alzheimer’s disease (AD), including APP/PS1 mice (Holtman et al., 2015; Krasemann et al., 2017; Kang et al., 2018; Guillot-Sestier et al., 2021), 5XFAD mice (Keren-Shaul et al., 2017) and App^*NL*–*G*–*F*^ mice (Sala Frigerio et al., 2019).

Analysis of metabolism in microglia has determined that stimulation of cultured cells from neonates with immune stimuli triggers the cells to shift their metabolism toward glycolysis accompanying the increase in expression of activation markers (Holland et al., 2018; Rubio-Araiz et al., 2018; Baik et al., 2019; Nair et al., 2019). The amyloid-β (Aβ) and lipopolysaccharide (LPS)-induced increases in glycolytic flux in cultured microglia (Baik et al., 2019; McIntosh et al., 2019) have been attributed to mTOR-HIF-1α pathway activation (Baik et al., 2019) or iron accumulation in microglia (McIntosh et al., 2019) and these changes translated into 2 models of AD, 5XFAD and APP/PS1 mice (Baik et al., 2019; McIntosh et al., 2019). Furthermore, microglia from aged mice (McIntosh et al., 2019; Mela et al., 2020) exhibited a similar reprogramming of metabolism to a glycolytic state. An important finding was that function, including phagocytosis and chemotaxis, was compromised in glycolytic cells (Mela et al., 2020; Guillot-Sestier et al., 2021) perhaps as a result of reduced ATP production, which would also be a consequence of mitochondrial dysfunction. This compromised function was supported by the finding that microglial recruitment to the site of laser injury was reduced in 5XFAD mice compared with WT mice, although the authors suggested that such immediate reactivity was dependent on glycolytic metabolism (Baik et al., 2019).

Many studies implicate microglia in the pathogenesis of AD but the exact mechanism(s) have not been settled. We have recently demonstrated that there is a sex-related difference in expression of genes linked with inflammation/immune activation in microglia from APP/PS1 mice, with increased expression in microglia from females compared with males (Guillot-Sestier et al., 2021). Furthermore, microglia from female APP/PS1 mice exhibited a glycolytic phenotype coupled with reduced phagocytic activity, whereas no marked changes were observed in cells from males. Whether this sex-related switch toward glycolysis is accompanied by a change in mitochondrial metabolism is not known but it is significant that mitochondrial damage has also been reported in microglia in APP/PS1 mice and that defective mitophagy has been associated with reduced clearance of Aβ (Fang et al., 2019).

Here we sought to assess whether there were sex-related differences in microglia that might reflect mitochondrial dysfunction in tissue from APP mice compared with WT mice. The data provide evidence of greater microglial dystrophy in sections from female AD patients compared with males, associated with similar sex-related differences in P16 and lipofuscin accumulation. Iron accumulation was also more marked in microglia from female AD patients compared with males and the presence of iron was particularly marked in spheroidal swellings which, together with beaded/discontinuous processes and rod-shaped microglia, provided further evidence of dystrophic microglia that were more prevalent in tissue from female AD patients. A similar sex-related difference in iron accumulation was observed in APP/PS1 mice and P16 and lipofuscin accumulation were also increased to a greater extent in microglia from female, compared with male APP/PS1 mice. A more marked downregulation of genes that code for proteins which support mitochondrial function were observed in microglia isolated from female APP/PS1 mice, as well as an increase in reactive oxygen species (ROS) production. These changes, together with the sex-related difference in mitochondrial metabolism, are consistent with perturbation of mitochondrial function and support the growing evidence that sex-related differences in microglia may contribute to the well-described sexual dimorphism in AD.

## Materials and Methods

### Animals

In this study, we used 17–18 month-old male and female transgenic mice that overexpress mutant amyloid precursor protein (APP_*swe*_) and presenilin 1 (PSEN1_Δ*E*9_; APP/PS1 mice, B6;C3-Tg (APP_*swe*_PSEN1_Δ*E*9_)85Dbo/Mmjax MMRRC (RRID:MMRRC_034829-JAX) and littermate controls. Mice were maintained under veterinary supervision, and experiments were conducted under license from the Health Products Regulatory Authority (HPRA), Ireland (Project license numbers AE19136/P035 and AE19136/P113) in accordance with EU regulations and with local ethical approval (Animal Research Ethics Committee, Trinity College Dublin). Mice were housed at 20–22°C, and had unlimited access to food and water.

### Human Samples

Paraffin-embedded human brain tissue was obtained from the Netherlands Brain Bank, which received consent from potential donors, having applied current legal and ethical guidelines for brain autopsy, tissue storage and use of tissue for scientific research worldwide. The experiments described here were undertaken with additional local ethical approval (Faculty of Health Sciences Research Ethics Committee, Trinity College Dublin). Analysis was undertaken on cortical tissue from 5 male and 4 female individuals, aged 75–82 years (mean ± SEM = 76.67 ± 1.41) with confirmed AD (Braak score = 5) and from 5 male and 4 female non-demented controls (aged 71–82; mean ± SEM = 77.89 ± 0.71; Braak score = 1 or 2).

### Immunohistochemical Analysis of Human Tissue

Sections of parietal cortex (12 μm) from post-mortem tissue of male and female AD patients and age-matched controls were heated (37^°^C, 3h) and incubated in xylene (2 × 15 min, Sigma-Aldrich) to deparaffinize. Tissue was rehydrated by incubating in ethanol (100% ×10 min, 90% × 10 min, 75% × 10 min) and dH_2_O (30 min) and endogenous peroxidase activity was quenched by incubating in H_2_O_2_ solution (1% in 20% methanol, 20 min, room temperature). Sections were rinsed in PBS and incubated with citrate-Na (pH 6.0) to facilitate antigen retrieval (2 × 5 min in microwave). After thorough washing in TBS (3 × 5min), sections were blocked in normal horse serum (20% in 0.2% Triton, 45 min) and incubated overnight in the presence of rabbit anti-Iba1 (1:1,000, Wako, Japan; 4°C in a humid tray chamber). Sections were washed with PBS and incubated (90 min) with biotinylated horse anti-rabbit secondary antibody, which was conjugated to HRP *via* the ABC method (Vector Laboratories). Staining was visualized with 3,3′-diaminobenzidine (DAB) as the chromogenic substrate.

Staining for free ionic iron (Fe^3+^) in Iba1-stained tissue sections was performed by Prussian blue histochemical reaction using the HEMATOGNOST Fe^®^, staining kit (Millipore, Ireland) as per the manufacturer’s instructions. In brief, sections were incubated 1:1 with potassium hexacyanoferrate (II) solution and hydrochloric acid (37°C, 20 min). Sections were rinsed in dH_2_O, dehydrated by incubating in ascending ethanol concentrations, cleared in xylene and mounted on coverslips using DPX mounting media.

We also assessed P16^*INK*4*a*^ and lipofuscin as measures of cell senescence. For p16^*INK*4*a*^ immunostaining, sections were pre-treated as above, immune-blocked in 20% NHS and incubated overnight in an anti-p16^*INK*4*a*^ monoclonal antibody (1:500, MA5-17142, Invitrogen). Sections were rinsed in PBS, incubated in a biotinylated horse anti-mouse secondary antibody and thereafter in an avidin-bound HRP conjugate. Staining was visualized with 3,3′-diaminobenzidine (DAB) as the chromogenic substrate.

Sudan Black B (SBB) was used as a stain for lipofuscin as previously described (Evangelou and Gorgoulis, 2017). In brief, sections were de-waxed, rehydrated, washed and incubated in citric acid buffer (pH 6.0; 2 × 5 min; in a microwave oven). Slide-mounted tissue sections were lowered onto a slide which was coated with freshly-prepared, filtered SBB solution (0.7 g in 100 ml 70% ethanol) and incubation proceeded (5 min, room temperature). Slides were then quickly immersed in 50% ethanol solution, washed in dH_2_O, sections were dehydrated briefly in 50% ethanol and mounted on coverslips using DPX mounting media.

### Image Acquisition and Analysis

For DAB immuno-peroxidase images, 12 images were taken from 3 sections per subject and analyzed in a blind, unbiased manner. Sections were imaged at 40x magnification using an Olympus DRP72 camera mounted on an Olympus BX51 light microscope. The number of dystrophic microglia (i.e., microglia exhibiting evidence of deramified branches, spheroids, beaded processes and rod-shaped cell somas) was quantified using the cell counter tool on ImageJ (National Institute of Health)^1^ and expressed as a percentage of total Iba1^+^ cells per field of view. For confocal immunofluorescence microscopy, sections were viewed using a Leica SP8 scanning confocal microscope and analysis was undertaken with ImageJ and Imaris software. To analyze iron-laden microglia the cell counter tool on ImageJ (National Institute of Health, see text footnote 1) was used to quantify the number of Iba1^+^iron^+^ cells, which was expressed as a percentage of total Iba1^+^ cells per field of view.

For analysis of p16^*INK*4*a*^, images were taken at 20x magnification, converted to 8-bit binary and a consistent threshold was set for all images. Images were pixel-filtered to remove background and p16^*INK*4*a*^-positive immunostaining was assessed using the Analyze particles tool. For analysis of SBB staining, images were taken at 40x magnification to visualize perinuclear and cytoplasmic intracellular aggregates of blue-black lipofuscin granules. Binary images were pixel-filtered and the % area of SBB-positive lipofuscin was measured using the Analyze particles tool on ImageJ.

### Preparation of Mouse Tissue

Mice were anaesthetized with sodium pentobarbital (Euthanimal), transcardially-perfused with saline and the brain was dissected free and used either to isolate microglia for immunocytochemistry, analysis of metabolic profile or gene expression analysis, or tissue for PCR or for immunohistochemistry (IHC). In the latter case, brains were perfused with cold PBS and PFA (4%), incubated in PFA (4%, 24 h) and stored in sucrose (30%). Coronal sections of cortex (40μm) were prepared and stored (30% ethylene glycol, 30% sucrose in PBS, –20°C) for later analysis.

### Preparation of Microglia

Isolated microglia were prepared from brain tissue as described (Mela et al., 2020). In brief, tissue was homogenized (Gentle-MACS Dissociator and the Adult Brain Dissociation Kit, Miltenyi Biotec, United Kingdom), filtered, washed in Dulbecco’s phosphate-buffered saline (D-PBS) containing calcium (100 mg/l), magnesium (100 mg/l), glucose (1,000 mg/l), pyruvate (36 mg/l) and centrifuged (3,000 × g, 10 min) to obtain supernatants. Samples were centrifuged (300 × g, 10 min) to yield a pellet that contained microglia which were resuspended in D-PBS, incubated with CD11b microbeads (Miltenyi Biotec. United Kingdom) and magnetically-separated using the QuadroMACS separator (Miltenyi Biotec, United Kingdom). Samples were resuspended in PBS containing 0.5% fetal bovine serum, centrifuged (300 g, 10 min) to provide a pellet that was resuspended in Dulbecco’s modified Eagle’s medium.

### Nanostring

RNA was isolated from microglia (400,000 cells/well in 12-well plates; final volume 800 ml) using the NucleoSpin RNA II kit (Macherey-Nagel, Duren, Germany) and stored at –80°C. Gene expression was evaluated in samples in nCounter hybridization reactions using the NanoString Reporter CodeSet, with added capture ProbeSet and hybridation buffer and Proteinase K according to the manufacturer’s instructions (nCounter Mouse Glial Profiling Panel). Samples were thawed, unamplified RNA (30 ng) was hybridized (overnight, 65°C in thermal cycler) and samples were loaded on a nCounter MAX Analysis system. Data analysis was performed using nSolver software; negative and positive controls were used to normalize for variability in hybridization, purification or binding. Raw data were normalized using the geometric mean of housekeeping genes).

### Analysis of Microglial Oxygen Consumption Rate

The SeaHorse Extracellular Flux Analyzer (SeaHorse Bioscience, United States) was used to analyze microglial metabolism as previously described (McIntosh et al., 2019). Microglia (50,000 cells/well; final volume 200 μl; 4–6 replicates/sample) were seeded on SeaHorse cell culture microplates, the sensor cartridge was hydrated by adding SeaHorse XF Calibrant solution (200 μl) to each well and samples were left overnight in a CO_2_-free incubator at 37°C. Cells were washed, assay media added to give a final volume of 200 μl/well and incubation continued (37°C; 1 h; CO_2_-free incubator). For the assay, oligomycin (20 μM; AbCam, United Kingdom), carbonyl cyanide-4-(trifluoromethoxy) phenylhydrazone (20 μM; FCCP; Sigma-Aldrich, United Kingdom) and antimycin A (40 μM; Sigma-Aldrich, United Kingdom) were loaded into the appropriate ports for sequential delivery at 24 min intervals and, following calibration, Oxygen Consumption Rate (OCR) was measured at 8 min intervals for a total of 96 min and automatically calculated using the SeaHorse XF96 software.

### Immunocytochemistry

The fluorescent probe, CellROX™ (Thermo Fisher Scientific, C10448), was used to assess ROS. Cells were washed with pre-warmed PBS, incubated in the presence of the CellROX™ Reagent (10 min, at 37°C; final concentration 5μM), washed, fixed (4% PFA, 15 min) and washed. Coverslips with the cells were mounted onto slides and stored at 4°C until imaging. Images of cells were taken using a Leica SP8 scanning confocal microscope (40X, 5 fields of view). CellROS expression in DAPI-stained nuclei and cytosol was assessed. ImageJ software (National Institute of Health, see text footnote 1) was used to convert images to 8-bit greyscale, a consistent threshold and mask were set and binarized images were filtered by pixel size to reduce background and enhance contrast. Mean Integrated Density (IntDen) was measured using the “analyze particles” tool.

### Immunohistochemical Analysis of Mouse Tissue Sections

For analysis of tissue from aged WT and APP/PS1 mice 4 images were taken from 2 sections per subject and analyzed in a blind, unbiased manner. Iba1 was used to stain microglia and iron was visualized by the Prussian blue reaction (Hematognost Fe, Millipore Ireland) (McIntosh et al., 2019). Sections were washed, blocked for endogenous peroxidase and non-specific binding by incubating in hydrogen peroxide (0.75%; 20 min) and normal horse serum (10%; 1 h), respectively, washed, incubated in rabbit anti-Iba1 primary antibody (WAKO, Japan; 1:5,000; 4°C; overnight), washed again and incubated in goat anti-rabbit biotinylated secondary antibody (Vector Labs, United Kingdom; 1:300; 90 min). Sections were processed using the avidin-biotin immunoperoxidase method (Vectastain ABC Elite kit, Vector Laboratories, United Kingdom) and Iba1 was visualized by the addition of the chromogen 3′,3-diaminobenzidine (containing 0.02% H_2_O_2_ in PBS, Sigma-Aldrich, United Kingdom). To assess iron, sections were incubated (1:1 potassium hexacyanoferrate (II; 4.78%) and HCl (4%); 20 min, 37°C), washed, dehydrated through graded alcohols, immersed in 100% xylene (VWR International United Kingdom) and mounted with DPX (Sigma). Samples were viewed on an Olympus BX51 light microscope with a built-in camera (Olympus, Japan) and iron^+^ Iba1^+^ cells were calculated as a proportion of the total number of Iba1^+^ cells per field of view. Congo red staining was used to visualize amyloid plaques (McIntosh et al., 2019).

For P16^*INK*4*a*^ staining, free-floating sections were incubated in PBS containing Triton X100 (0.3%; 5 min), washed, incubated in blocking buffer (PBS containing saponin (0.1%) and goat serum (2%); room temperature; 1 h). The sections were incubated with rabbit anti-Iba1 primary antibody and mouse anti-p16^*INK*4*a*^ antibody (WAKO, Japan; 1:1,000; Thermo Fisher Scientific, Warrington, United Kingdom; 1:1,000; 4°C; 48 h). Samples were washed and incubated in the secondary antibodies (Alexa Fluor 488 goat anti-rabbit, 1:1,000; Alexa Fluor 594 goat anti-rabbit, 1:1,000, Thermo Fisher Scientific, Warrington, United Kingdom; 4°C; 24 h). Sections were washed, mounted with Vectashield containing DAPI, coverslipped, sealed and stored at 4°C. Images were taken using the Leica Biosystems SP8 scanning confocal microscope in 3 dimensions and analyzed using Imaris software (Oxford Instruments, United Kingdom). A 3D surface was created using the surface tool for the Iba1 and P16 signals and the volume of P16 within Iba1^+^ cells was determined and normalized to the number of Iba-1^+^ cells per image.

As a measure of lipofuscin accumulation in Iba1^+^ microglia, we assessed autofluorescence in tissue free-floating sections from the 4 groups of mice. To stain for Iba1, sections were incubated in PBS containing Triton ×100 (0.3%; 5 mins), incubated in blocking buffer (PBS containing Triton ×100 (0.3%) and goat serum (2%); room temperature; 1 h). Sections were incubated with rabbit anti-Iba1 primary antibody (WAKO, Japan; 1:1,000; 4°C; 48 h), washed and incubated with the secondary antibody, Alexa Fluor 647 goat anti-rabbit IgG (Thermo Fisher Scientific, Warrington, United Kingdom, 1:1,000; 4°C; 24 h), washed, mounted and stored at 4°C. Images were taken using the Leica Biosystems SP8 scanning confocal microscope in 3 dimensions, using the excitation wavelength 488 nm to capture the autofluorescence. The images were analyzed using Imaris Software as described above for P16.

### Analysis of Inflammatory Cytokines by PCR

mRNA expression of IL-1β, TNFα, and IL-6 was assessed in samples of hippocampal homogenate. RNA was isolated from microglia using the Nucleospin RNAII KIT (Macherey-Nagel, Duren, Germany) and cDNA was prepared using High-Capacity cDNA RT kit according to the manufacturer’s instructions (Applied Biosystems, United Kingdom). Real-time PCR was performed with predesigned Taqman gene expression assays (IL-1β (Mm00434228_m1); TNFα (Mm00443258_m1); IL-6 (Mm00446190_m1); Applied Biosystems, United Kingdom) using an Applied Biosystems 7500 Fast Real-Time PCR machine (Applied Biosystems, Germany). Samples were assayed as previously described (Costello et al., 2016) with β-actin (Mm00407939_s1) as the endogenous control to normalize gene expression data. Gene expression was calculated relative to the endogenous control samples and to the control sample giving an RQ value (2^– DDCt^, where Ct is the threshold cycle).

### Statistical Analysis

Data are reported as the mean ± SEM and the number of experiments is indicated in each case. Statistical analysis was carried out using a two-way analysis of variance (ANOVA), with *post hoc* Tukey’s multiple comparisons tests. The significance level was set at *p* < 0.05.

## Results

**FIGURE 1:**
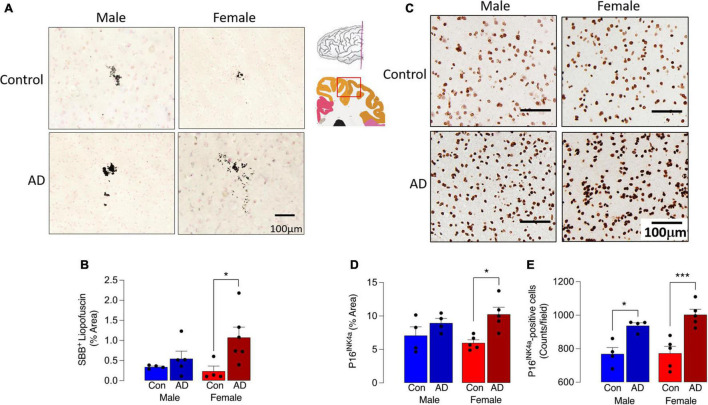
Sex-specific increase in senescent cells in AD. **(A)** Sudan Black B staining of lipofuscin was increased in sections from AD patients compared with age-matched controls. **(B)** A significant main effect of disease was observed (*p* < 0.05), and *post hoc* analysis indicated that staining was significantly greater in sections from female AD patients compared with controls (*p < 0.05). **(C,D)** P16^*INK*4*a*^ staining was most pronounced in tissue from female AD patients and analysis of the data indicated that there was a significant main effect of disease (*p* < 0.01) with *post hoc* analysis establishing that staining was significantly greater in sections from female AD patients compared with female controls (*p < 0.05). **(E)** The number of P16^*INK*4*a*^-stained cells was significantly increased in sections from AD patients compared with control (*p* < 0.001; significant main effect of disease) with *post hoc* analysis establishing that staining was significantly greater in sections from female AD patients compared with female controls and in sections from male AD patients compared with male controls (*p < 0.05; ***p < 0.001). The inset shows the region from which sections were prepared. Data (mean ± SEM; *n* = 3–5) represent the mean area stained for Sudan Black B or P16^*INK*4*a*^ expressed as a% of the total.

We first assessed 2 markers of cell senescence, lipofuscin and P16, in sections of parietal cortex from postmortem brain of male and female AD patients and age-matched controls. Lipofuscin, an aggregate of oxidized proteins, lipids, and metals, formation of which is perhaps triggered by mitochondrial and/or lysosomal dysfunction (Terman and Brunk, 2006; Konig et al., 2017), accumulates in senescent cells including microglia (Safaiyan et al., 2016; O’Neil et al., 2018). Here we show that Sudan Black B staining of lipofuscin was increased in tissue from AD patients compared with controls (Figure 1A) although the cell type associated with this increase was not assessed. Analysis of the data indicated that there was a main effect of disease (*p* < 0.05), while *post hoc* analysis indicated that staining was significantly greater in sections from female AD patients compared with female controls (*p < 0.05; Figure 1B). No difference between male AD patients and controls was observed. P16 accumulation, which correlates with other makers of senescence, has been shown to increase as early as 4 months of age in hippocampus of the MAPT P301S PS19 mouse model of tau-dependent neurodegenerative disease (Bussian et al., 2018). Here we show that P16 staining was markedly more pronounced in brain sections from AD patients (*p* < 0.01; Figure 1C) compared with controls and this may be attributable to the AD-related increase in the number of P16^+^ cells (*p* < 0.001). We did not assess the cell type associated with this increase. *Post hoc* analysis revealed that staining was significantly greater in sections from female AD patients compared with female controls (*p < 0.05) but not males (Figure 1D), whereas P16^+^ cell number was increased in female AD patients compared with female controls (*p < 0.001) and also in male AD patients compared with male controls (*p < 0.05).

**FIGURE 2:**
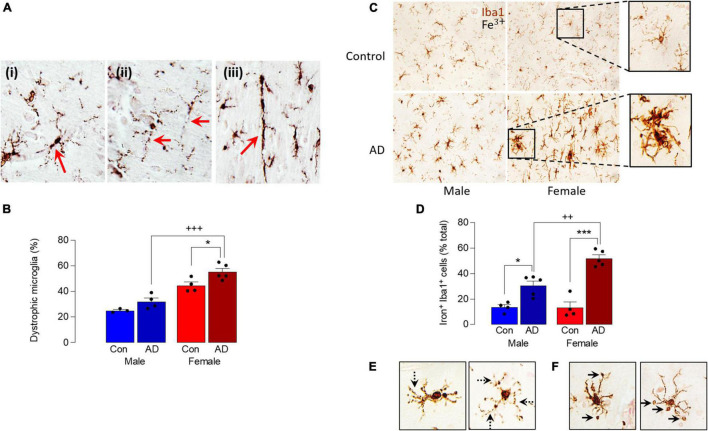
Sex-specific increase in dystrophic and iron-laden microglia in AD. **(A)** Analysis of microglial morphology in postmortem tissue (parietal cortex) from male and female AD patients and age-matched controls revealed microglia with fragmented soma and processes (i), beading in the processes (ii) and a distinctive rod shape (iii). **(B)** Quantitative analysis of these microglial features identified a significant main effect of sex (*p* < 0.001) and disease (*p* < 0.01) and *post hoc* analysis indicated a significant increase in dystrophic microglia in tissue from female AD patients compared with controls (*p < 0.05) and male AD patients (^+++^*p* < 0.001). Data are expressed as the mean (± SEM; *n* = 3–5) dystrophic microglia as a percentage of the total number of microglia **(C)**. Iron staining was increased in Iba1^+^ microglia in sections from AD samples compared with age-matched controls, particularly in female AD patients. **(D)** A significant main effect of sex (*p* < 0.05) was observed and *post hoc* analysis indicated that the percentage of iron^+^ Iba1^+^ cells was significantly increased in tissue from male and female AD patients compared with their respective controls (*p < 0.05; ***p < 0.001), and in female AD patients compared with male AD patients (^++^*p* < 0.01). Data (means ± SEM; *n* = 4–5) are Iron^+^ Iba1^+^ cells expressed as a % of the total Iba1^+^ cells. **(E,F)** Iron accumulation was evident in dystrophic cells **(E)** and in spheroids **(F)**.

A comparison of Iba1^+^ microglia in sections of parietal cortex from postmortem brain of male and female AD patients and age-matched controls indicated an increase in dystrophic cells, typified by evidence of fragmentation and beading of cytoplasmic processes, densely-stained spheroid-shaped process endings (Figure 2A). Specifically, we observed microglia with processes that had a fragmented appearance (Figure 2Ai) and evidence of beading (ii), and we also observed many rod-shaped microglia (iii). Analysis of the mean data obtained from dystrophic cell counts indicated that there was a significant main effect of sex (*p* < 0.001) and disease (*p* < 0.01; Figure 2B) and *post hoc* analysis indicated that there was an increase in dystrophic microglia in tissue from female AD patients compared with female controls (*p < 0.05) and compared with male AD patients (^+++^*p* < 0.001). There was also a significantly greater proportion of dystrophic microglia in tissue from female controls compared with male controls (*p* < 0.01). The distribution of dystrophic cells was roughly homogenous in the images analyzed.

These data suggest that microglia are markedly affected by sex in AD and, because iron handling by cells is inextricably linked with altered microglial phenotype (McIntosh et al., 2019; Ndayisaba et al., 2019), we examined iron accumulation in Iba1^+^ microglia in sections from male and female AD patients and controls. The data show that iron staining was increased in microglia from AD samples compared with controls and markedly enhanced in samples from female AD patients (Figure 2C). Analysis of the quantitative data indicated a significant main effect of sex (*p* < 0.05) and *post hoc* analysis indicated that the percentage of iron^+^ Iba1^+^ cells was significantly increased in male AD patients compared with male controls (*p < 0.05; Figure 2D), in female AD patients compared with female controls (^***^p < 0.001); staining was also significantly increased in sections from female AD patients compared with sections from male AD patients (^++^*p* < 0.01). Further analysis of the images indicated that iron accumulation was evident in dystrophic cells that were characterized by beaded and fragmented processes (Figure 2E), and that it was especially concentrated in spheroids (Figure 2F). The changes are associated with accumulation of amyloid, which we have previously reported is increased to a greater extent in cortex from female AD patients compared with males (Guillot-Sestier et al., 2021); here we show a similar sex-related difference in hippocampus (Supplementary Figure 1).

**FIGURE 3:**
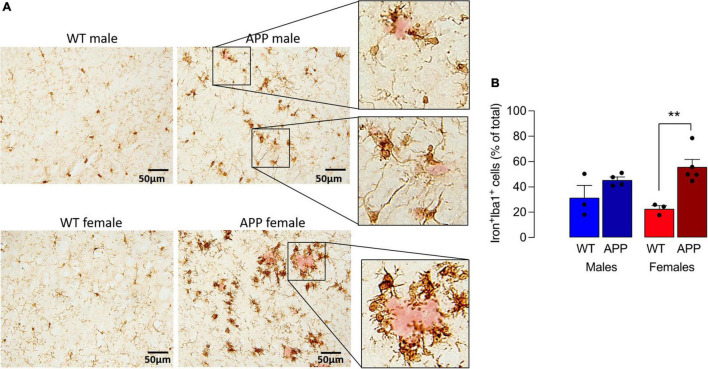
Iron accumulation in microglia is increased to a greater extent in sections from female APP/PS1 mice. **(A)** Iron staining was increased in Iba1^+^ microglia in sections from APP/PS1, compared with WT, mice notably in sections from females. Iron^+^ Iba1^+^ cells clustered around (pink-stained) Ab-containing plaques (insets). **(B)** A significant main effect of genotype was observed (*p* < 0.01) and *post hoc* analysis revealed that the proportion of Iron^+^ Iba1^+^ cells was significantly greater in sections of female APP/PS1 mice compared with female WT mice (**p < 0.01). Data (means ± SEM; *n* = 3–5) are Iron^+^ Iba1^+^ cells expressed as a % of the total Iba1^+^ cells.

To explore this further, we turned to the APP/PS1 mouse model of AD and show that iron staining in microglia was markedly greater in sections from APP/PS1 mice compared with WT mice and the most marked staining was in female APP/PS1 mice where the iron-labeled cells seemed to cluster around plaques (stained pink; Figure 3A). Analysis of the quantitative data indicated that there was a main effect of genotype on the proportion of iron^+^ Iba1^+^ cells (*p* < 0.01) and *post hoc* analysis revealed that there was a significant difference between female WT and female APP/PS1 mice (^**^p < 0.01; Figure 3B). Previous findings have indicated that iron accumulation in microglia triggers production of inflammatory cytokines (McIntosh et al., 2019). Here, we confirm this and report that there was a significant sex x genotype interaction for IL-1 β (*p* < 0.01), TNFα and IL-6 (*p* < 0.05; Supplementary Figure 2) in hippocampal tissue prepared from WT and APP/PS1 male and female mice and a significant increase (^**^p < 0.01; ^***^p < 0.001) in tissue from APP/PS1 females compared with WT females and between APP/PS1 males and females (^++^*p* < 0.01; ^+++^*p* < 0.001).

**FIGURE 4:**
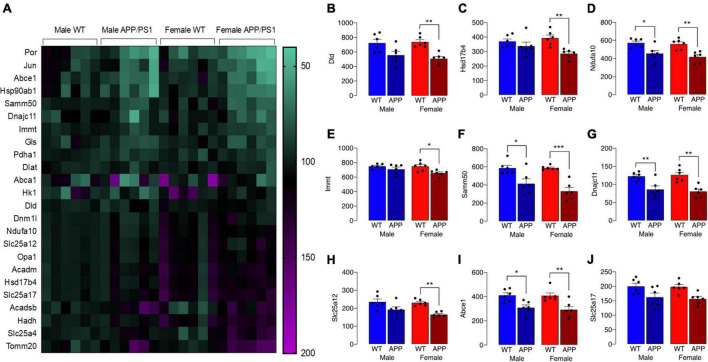
Downregulation of some gene transcripts that code for mitochondrial proteins is sex-specific. **(A)** The heat map of data from Nanostring analysis indicates that transcripts of genes coding for mitochondrial proteins were downregulated (depicted in green) to a greater extent in microglia from female APP/PS1 mice compared with other groups, whereas there was little evidence of marked upregulation (depicted in pink/purple) of genes. **(B–J)** A significant main effect of genotype (*p* < 0.01 for *Hsd17b4, Immt, Abce1*, and *Slc25a17; p* < 0.001 for all others; 2 way ANOVA) was identified and *post hoc* analysis indicated that there were significant decreases in most transcripts in cells from female APP/PS1 mice compared with female WT mice (*p < 0.05; **p < 0.01; ***p < 0.001) and significant decreases in *Ndfua10, Samm50, Dnajc11*, and *Abce1* in cells from male APP/PS1 mice compared with male WT mice (*p < 0.05; **p < 0.01). Data are expressed as the mean ± SEM (*n* = 6).

**FIGURE 5:**
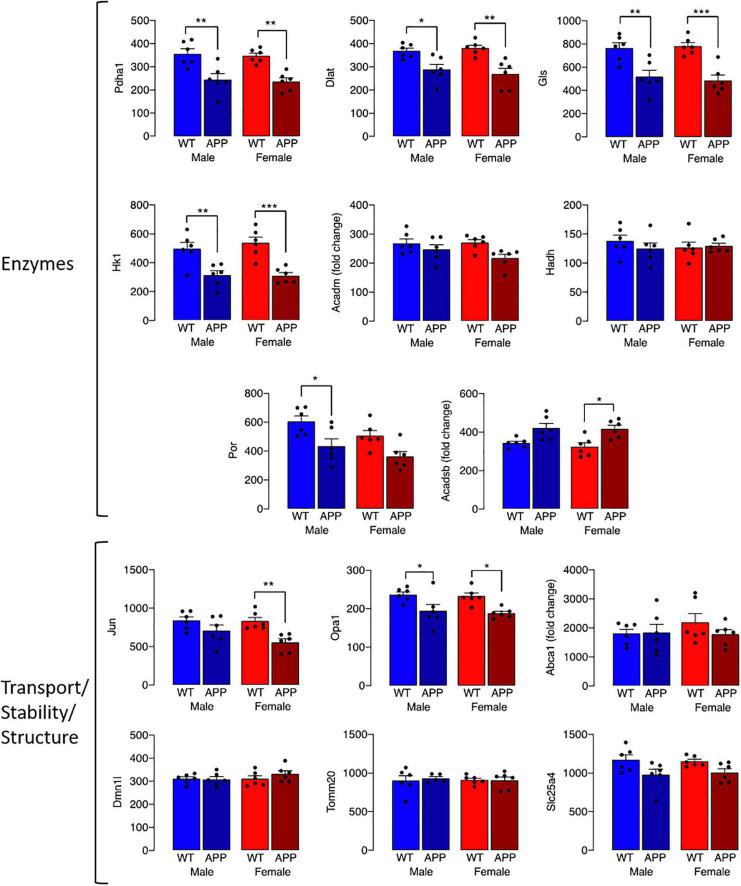
Genotype-related changes in gene transcripts that code for mitochondrial proteins. Genotype-related changes were identified in transcripts of genes that code for mitochondrial enzymes, *Pdha1, Dlat, Gls, Hk1, Por, Acadsb* (*p* < 0.001), *Acadm* (*p* < 0.05) and genes that code for mitochondrial transport and stability like *Opa1, Slc25a4* (*p* < 0.001), *Jun* (*p* < 0.01) and *Por* (*p* < 0.05). *Post hoc* analysis revealed significant genotype-related differences in male APP/PS1 mice compared with male WT mice and female APP/PS1 mice compared with female WT mice (*p < 0.05; **p < 0.01; ***p < 0.001). Data are expressed as the mean ± SEM (*n* = 6).

The observed change in iron accumulation suggests an alteration in mitochondrial function and, to investigate this further, we assessed transcripts of genes that play a role in mitochondrial function. Data from Nanostring analysis indicate that transcripts were downregulated (green) to a greater extent in microglia from female APP/PS1 mice compared with other groups (Figure 4A). Genotype-related changes were identified in transcripts of genes that code for mitochondrial enzymes like *Dld, Hsd17b4* and *Ndufa10*, or proteins that play a role in maintenance of mitochondrial membrane structure like *Immt*, *Samm50* and *Dnajc11*, or modulate transport like *Slc25a12, Abce1* and *Slc25a17* (Figures 4B–J). Analysis revealed a significant main effect of genotype (*p* < 0.01 for *Hsd17b4, Immt, Abce1*, and *Slc25a17; p* < 0.001 for all others; 2 way ANOVA). *Post hoc* analysis indicated sex-specific significant decreases in *Dld, Hsd17b4*, and *Slc25a12* (*p < 0.05; ^**^p < 0.01) in cells from female APP/PS1 mice compared with WT mice and significant genotype-related decreases in other transcripts in both males and females (*p < 0.05; ^**^p < 0.01). Analysis of other changes (Figure 5) identified a genotype-related downregulation of transcripts that code for mitochondrial enzymes *Pdha1, Dlat, Gls, Hk1, Por, Acadsb* (*p* < 0.001) and genes that code for mitochondrial transport and stability like *Opa1, Slc25a4* (*p* < 0.001), *Jun* (*p* < 0.01) and *Por* (*p* < 0.05). *Post hoc* analysis revealed significant genotype-related differences in male APP/PS1 mice compared with male WT mice and female APP/PS1 mice compared with female WT mice (*p < 0.05; ^**^p < 0.01; ^***^p < 0.001). In contrast to other transcripts which were downregulated or showed no change, *Acadm* was upregulated with evidence of a significant main effect of genotype (*p* < 0.05) and a significant difference between female APP/PS1 mice compared with female WT mice (*p < 0.05).

**FIGURE 6:**
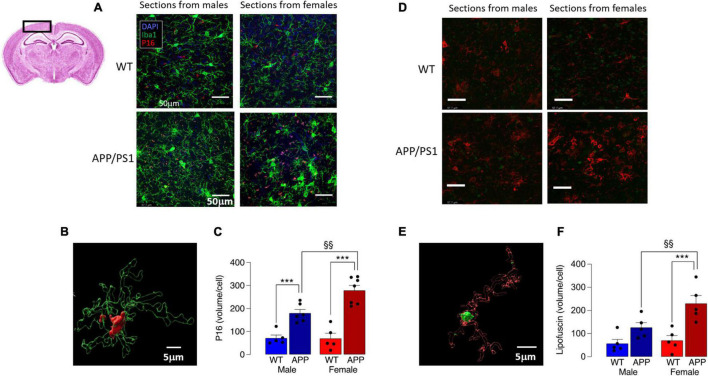
Increased P16 accumulation in microglia from female APP/PS1 mice suggests sex-related cell senescence. **(A)** Staining of sections of cortical tissue (see inset) with P16 indicates that accumulation of this marker of cell senescence was greater in Iba1^+^ microglia from female APP/PS1 mice compared with cells from other groups. **(B)** A 3D image, was created using the surface tool in Imaris, for the Iba1 and P16 identifies intracellular P16 and permits calculation of P16 volume within microglia. **(C)** A significant genotype x sex interaction was observed (*p* < 0.05) and *post hoc* analysis indicated that there was a significantly greater P16 accumulation (expressed as μm^3^ corrected for cell number) in microglia in sections from male and female APP/PS1 mice compared with their WT counterparts (***p < 0.001) and significantly greater accumulation in cells from female APP/PS1 mice compared with male APP/PS1 mice (§§ *p* < 0.01). **(D)** Lipofuscin accumulation in sections of cortex was greater in Iba1^+^ microglia from female APP/PS1 mice compared with the other groups. **(E)** A 3D image identifies lipofuscin accumulation (autofluorescence at excitation 488 nm) in an Iba1^+^ microglial cell. **(F)** Significant effect of genotype (*p* < 0.001) and sex (*p* < 0.05) interaction was observed and *post hoc* analysis indicated that lipofuscin accumulation was significantly greater in microglia from female APP/PS1 mice compared with female WT mice (***p < 0.001) and male APP/PS1 mice (§§ *p* < 0.01). Data are the means ± SEM of cells (4 replicates) prepared from 5 mice.

Mitochondrial dysfunction is associated with cell senescence, one marker of which is P16 (Chapman et al., 2019). Analysis of P16 accumulation indicated a marked increase in P16 in Iba1^+^ microglia in cortical sections from female APP/PS1 mice compared with the other groups (Figures 6A,B), with a significant genotype x sex interaction (*p* < 0.05) and significantly more P16 in microglia from APP/PS1 mice compared with WT mice (^***^p < 0.001; Figure 6C) for males and females. The data also show that P16 accumulation in Iba1^+^ cells in cortical sections from female APP/PS1 mice was significantly greater than male APP/PS1 mice (§§ *p* < 0.01), which is consistent with the apparently greater mitochondrial disruption in microglia from these mice. Assessment of lipofuscin accumulation in Iba1^+^ microglia revealed significant main effects of genotype (*p* < 0.001) and sex (*p* < 0.05; Figures 6D,E) and *post hoc* analysis reflected changes that resembled those in P16. Thus there was significantly greater lipofuscin staining in microglia in sections from female APP/PS1 mice compared with female WT mice (^***^p < 0.001; Figure 6F) and in microglia from female APP/PS1 mice compared with male APP/PS1 mice (§§ *p* < 0.01).

**FIGURE 7:**
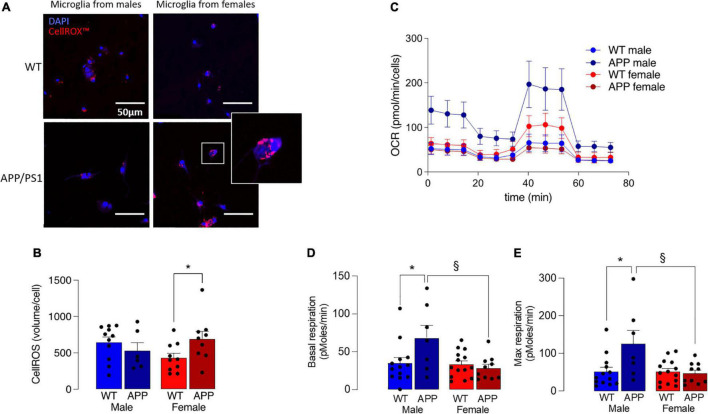
Mitochondrial disruption is more marked in microglia from female APP/PS1 mice. **(A)** Microglial staining with the fluorescent probe, CellROX™ is more marked in cells from female APP/PS1 mice compared with cells from other groups. **(B)** A significant genotype x sex interaction in CellROX™ was observed (*p* < 0.05) and *post hoc* analysis indicated that there was a significantly greater accumulation in cells from female APP/PS1 mice compared with female WT mice (*p < 0.05). Data are the means ± SEM of cells (4 or 5 replicates) prepared from 6 to 11 mice. **(C)** Mitochondrial metabolism, assessed by monitoring OCR over time, indicated increased metabolism in cells from male APP/PS1 mice compared with cells from the other 3 groups. **(D,E)** A significant main effect of sex was observed in basal respiration and maximal respiration (*p* < 0.05). *Post hoc* analysis indicated that basal and maximal respiration were significantly increased in microglia from male WT mice compared with male APP/PS1 mice (*p < 0.01) and significantly decreased in microglia from female APP/PS1 mice compared with male APP/PS1 mice (§ *p* < 0.01). Data are the means ± SEM of analysis from between 9 and 35 cell preparations obtained from 2 to 7 mice.

Increased production of ROS also accompanies disruption in mitochondrial dysfunction and, here, this was assessed by staining isolated microglia with the fluorescent probe, *CellROX*™. A significant genotype x sex interaction was found (*p* < 0.05; Figures 7A,B) and *post hoc* analysis indicated that there was a significantly greater accumulation of ROS in cells prepared from female APP/PS1 mice compared with female WT mice (§ *p* < 0.05; Figure 7B), whereas no genotype-related change was observed in cells prepared from male mice.

Microglia that produce inflammatory cytokines are glycolytic (Rubio-Araiz et al., 2018; McIntosh et al., 2019; Mela et al., 2020), opening the possibility that mitochondrial metabolism may also be affected. To directly assess this, we measured OCR in microglia isolated from the 4 groups of mice and show that it was decreased in microglia from female mice (Figure 7C), and significantly decreased in female APP/PS1 mice compared with male APP/PS1 mice (*p* < 0.01) and significantly decreased in male WT mice compared with male APP/PS1 mice (*p* < 0.01). A significant genotype x sex interaction was observed in basal and maximal respiration (*p* < 0.05) and *post hoc* analysis indicated that both measures were significantly increased in microglia from male WT mice compared with male APP/PS1 mice (*p < 0.01; Figures 7D,E) and significantly decreased in microglia from female APP/PS1 mice compared with male APP/PS1 mice (§ *p* < 0.01).

## Discussion

The key finding here is that there is a sex-dependent disruption in microglia in AD with evidence of cell dystrophy and senescence, associated with accumulation of iron and lipofuscin; these data cumulatively point to mitochondrial dysfunction, particularly in females. These findings are backed up by evidence from APP/PS1 mice in which the sex-related difference in iron accumulation is accompanied by evidence of ROS production and altered mitochondrial metabolism.

It has been known for almost 2 decades that microglial dystrophy, typified by beaded and discontinuous processes is a characteristic of the aged and Alzheimer brain (Streit, 2004). Here, we report a sex-related dimension with evidence of greater fragmented, beaded processes in tissue from female AD patients compared with males. There was also an increase in the number of rod-shaped microglia in tissue from female AD patients, paralleling previous similar observations in female APP/PS1 mice (Guillot-Sestier et al., 2021). Consistently, staining of P16, which identifies senescent cells, was also greater in tissue from female AD patients compared with males.

Iron accumulation, which is a feature particularly of dystrophic microglia (Lopes et al., 2008) is a characteristic of the AD brain (Bulk et al., 2018). It has been reported that it closely correlates with amyloid pathology (van Duijn et al., 2017), infiltrates Aβ-containing plaques (Kenkhuis et al., 2021) and contributes to amyloidosis (Bush, 2003) while iron^+^ cells cluster around Aβ plaques in tissue from AD patients (Meadowcroft et al., 2015), as they do in tissue from APP/PS1 mice (McIntosh et al., 2019). The present findings add to this evidence. First, we show that iron accumulation was clearly greater in Iba1^+^ microglia in sections from AD patients compared with controls and it was particularly marked in spheroidal swellings. Secondly, we demonstrate that there were significantly more iron^+^ Iba1^+^ cells in tissue from female AD patients compared with males and the same was the case in the animal model where the proportion of iron^+^ Iba1^+^ cells was increased in sections prepared from female APP/PS1 mice compared with male APP/PS1 mice. Iron distribution across cells is modulated by expression of import and export proteins, primarily divalent metal transporter1 and the export protein, ferroportin, and also storage proteins like ferritin (Song et al., 2010; Soares and Hamza, 2016). While this has not been assessed here, our previous data have indicated that iron retention is coupled with decreased ferroportin and increased ferritin which occur in the brain of APP/PS1 mice (Holland et al., 2018; McIntosh et al., 2019). However, other factors may also contribute to iron accumulation including increased blood brain permeability and inflammatory mediators (Ward et al., 2014) which have been reported in APP/PS1 mice (Minogue et al., 2014).

Mitochondrial function is closely associated with iron handling by cells (Ndayisaba et al., 2019) and it has been shown that microglia in aged animals accumulate lipofuscin, which comprises cross-linked proteins and lipids resulting from incomplete lysosomal digestion (Sierra et al., 2007). We now report a sex-related difference in lipofuscin accumulation in sections of cortex from AD patients compared with controls and a similar sex-related difference was observed in P16, both of which point to cell senescence. We further show that greater iron accumulation was increased in Iba1^+^ microglia in tissue from female AD patients. Therefore while staining for these 3 markers is similarly increased in females, we were unable to co-stain lipofuscin with Iba1 or P16 with Iba1, and therefore we cannot confirm that these changes were confined to microglia. However, similar links between P16 staining, lipofuscin accumulation and iron accumulation were observed in tissue from APP/PS1 mice and, in this case, the staining was assessed in Iba1^+^ cells, establishing that the changes were more marked specifically in microglia from female APP/PS1 mice compared with males. Together these data provide compelling evidence of greater tissue, and specifically microglial, senescence in female AD patients compared with males that is paralleled by changes in APP/PS1 mice.

We have previously shown that iron accumulation in microglia triggers the cells to produce inflammatory cytokines (Holland et al., 2018; McIntosh et al., 2019), which is confirmed here and extended to reveal a sex-related dimension. Specifically, the increased mRNA expression of IL-1β, TNFα and IL-6 are specifically identified in microglia from female APP/PS1 mice while there are minimal genotype-related changes in cells from male APP/PS1 mice, supporting previous data that demonstrated a preferential increase in markers of microglial activation in 9 month-old APP/PS1 female mice (Gallagher et al., 2013). In a previous study we used Nanostring technology to assess sex-related changes in expression of genes that have been used to describe so-called disease-associated microglia and activated response microglia (Keren-Shaul et al., 2017; Sala Frigerio et al., 2019), many of which reflect neuroinflammation/upregulated immune function. We reported that expression of several of these genes were particularly increased in microglia from female APP/PS1 mice, as were changes in genes that code for proteins involved in oxidative stress (Guillot-Sestier et al., 2021). These findings are also consistent with the sex bias in upregulation of genes that reflect inflammation and NFκB activation (Guillot-Sestier et al., 2021). Interestingly, single nucleus RNA sequencing has identified AD-related sex differences with evidence that genes reflecting microglial activation were enhanced in samples from females compared with males (Mathys et al., 2019) while transcriptomic analysis indicated that the age-related upregulation in microglia-specific genes that are indicative of inflammation occurs earlier in female, compared with male, mice (Mangold et al., 2017). At this point, there is widespread agreement that altered microglial profile is one of several changes in AD and models of AD that drives pathology (Leng and Edison, 2021) and, although the mechanism involved is not clear, this viewpoint is being consolidated by the accumulating evidence and points to sex-specific differences.

In the past few years, it has become clear that macrophages (Kelly and O’Neill, 2015) and microglia (McIntosh et al., 2019; Mela et al., 2020) which produce inflammatory molecules, become glycolytic. This is the case in microglia isolated from APP/PS1 mice and our recent work revealed that the shift toward glycolysis is significantly greater in microglia from female APP/PS1 mice compared with males, negatively impacting on microglial function (Guillot-Sestier et al., 2021). Metabolic disturbance in microglia from AD patients has also been demonstrated in a recent proteomic study which reported changes that are consistent with increased glycolysis (Johnson et al., 2020).

Mitochondrial dysfunction in AD is well documented (Wang et al., 2020) and decreased expression of proteins involved in oxidative metabolism has been reported in the brains of AD patients (Minjarez et al., 2016). Mitochondrial damage has also been reported in microglia in APP/PS1 mice and the accompanying defective mitophagy was associated with reduced clearance of Aβ and with increased production of inflammatory cytokines (Fang et al., 2019). The evidence from the present study also points to mitochondrial disruption in microglia from APP/PS1 mice and specifically shows a sex-related decrease in genes that code for mitochondrial proteins including enzymes like *Hsd17b4* and *Ndufa10*, transporters like *Slc25a12* and *Abce1*, and proteins involved in mitochondrial membrane architecture like *Opa1* and *Samm50* and *Immt*. This was paralleled by a sex-related difference in oxidative metabolism such that OCR was decreased particularly in microglia from female APP/PS1 mice. Interestingly, microglial metabolism, and specifically ATP production, was decreased in microglia from *TREM2*^–/–^ 5 × FAD mice although sex-related differences were not assessed (Ulland et al., 2017) and this finding is largely consistent with the present data.

Oxidative metabolism impacts on cell function and, in the present context, it is important to note that strategies which reduce oxidative phosphorylation decrease Aβ phagocytosis in 5 × FAD mice while strategies that increase it have the opposite effect (Pan et al., 2019). The present observation that oxidative metabolism is compromised microglia from female APP/PS1 mice together with our previous result indicating a shift toward glycolysis in these cells, provide a plausible explanation for the decrease in phagocytosis in microglia of APP/PS1 mice, particularly females, contributing to the sex-related increase in amyloidosis (Gallagher et al., 2013; Guillot-Sestier et al., 2021).

Disruption in mitochondrial function in microglia is associated with several changes including ROS production (Simpson and Oliver, 2020) and cell senescence (Chapman et al., 2019). A further indication of a sex-specific alteration in mitochondrial function is identified here since ROS was specifically increased in microglia from female APP/PS1 mice compared with WT whereas there was no genotype-related change in males, while P16 accumulation, which increases as early as 4 months of age in hippocampus of the MAPT P301S PS19 mouse model of tau-dependent neurodegenerative disease (Bussian et al., 2018), was also increased to a greater extent in microglia from female APP/PS1 mice compared with males.

To date, research has focused to a significant extent on assessing disease-related mitochondrial function in neurons (Fang et al., 2019; de la Fuente-Munoz et al., 2020; Messina et al., 2020), with limited direct analysis of changes in mitochondrial function in microglia. Therefore these data are important because they provide some explanation for the compromised microglial function that occurs with age in these mice (Guillot-Sestier et al., 2021).

Why sex-related differences in microglial phenotype exist in AD and APP/PS1 mice remains unclear but factors that are likely contributors include sex hormones, microRNA, chromosomal make-up and blood brain barrier permeability (Weber and Clyne, 2021; Lynch, 2022). For instance, neuromodulatory effects of estrogens have been described (Acosta-Martinez, 2020) and microRNAs, which also modulate immune networks, (Kodama et al., 2020) exhibit sex-related differences.

Genetic, hormonal and psychosocial factors are among those proposed as potential explanations for the sexual dimorphic nature of AD but the potential contribution of a shift in microglial phenotype to one that leads to production of inflammatory mediators is also being considered (Fisher et al., 2018). The evidence presented highlights marked sex-related differences in microglia in AD and in a model of the disease consistent with the growing evidence that microglial dyshomeostasis is a significant driver of pathology and may be a key factor in understanding the risk that sex poses in AD.

## Data Availability Statement

The datasets presented in this study can be found in online repositories. The name of the repository and accession number can be found below: National Center for Biotechnology Information (NCBI) Gene Expression Omnibus (GEO), https://www.ncbi.nlm.nih.gov/geo/, GSE203202.

## Ethics Statement

The studies involving human participants were reviewed and approved by the Faculty of Health Sciences Research Ethics Committee, Trinity College Dublin, Ireland. The Ethics Committee waived the requirement of written informed consent for participation.

## Author Contributions

EO’N, VM, AG, SB, AMcG, AW, and AMcI undertook experiments and analyzed data. ML designed and supervised the work. EO’N, VM, SB, and ML participated in preparing the manuscript. All authors contributed to the article and approved the submitted version.

## Conflict of Interest

The authors declare that the research was conducted in the absence of any commercial or financial relationships that could be construed as a potential conflict of interest.

## Publisher’s Note

All claims expressed in this article are solely those of the authors and do not necessarily represent those of their affiliated organizations, or those of the publisher, the editors and the reviewers. Any product that may be evaluated in this article, or claim that may be made by its manufacturer, is not guaranteed or endorsed by the publisher.

## Footnotes

1
https://imagej.nih.gov/ij/
