# Supplementary figures and images for: Sex-Related Microglial Perturbation Is Related to Mitochondrial Changes in a Model of Alzheimer’s Disease
Source: Front Cell Neurosci. 2022 Jul 5;16:939830. doi: 10.3389/fncel.2022.939830 (PMC9297004; doi:10.3389/fncel.2022.939830)

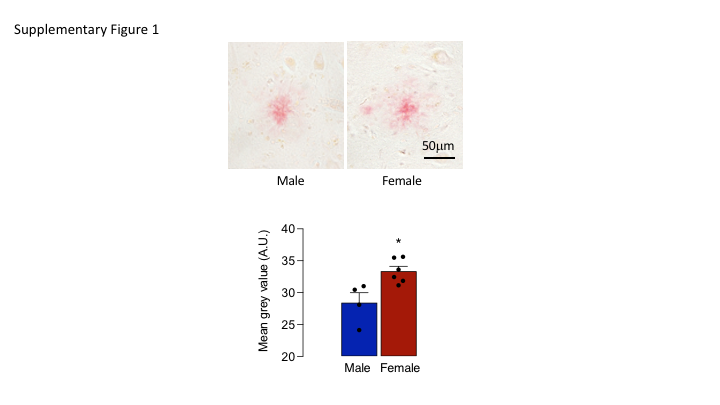

Supplement: Supplementary file 1 [file Figure_1.TIFF]

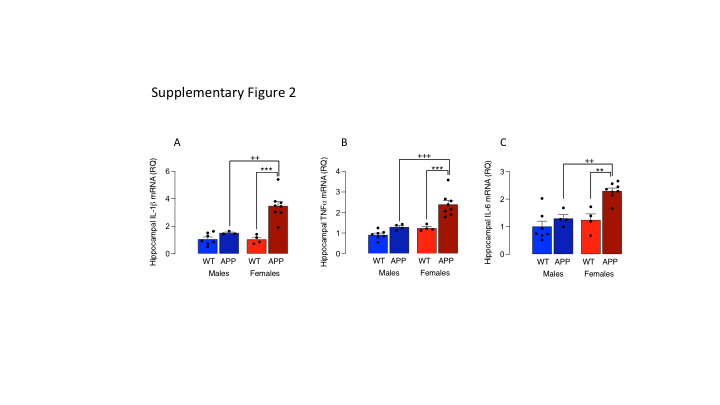

Supplement: Supplementary file 2 [file Figure_2.TIFF]
